# Suicidality among children and adolescents “in the COVID-19 era”: a worldwide metanalytic picture

**DOI:** 10.1093/eurpub/ckac131.492

**Published:** 2022-10-25

**Authors:** M Bersia, E Koumantakis, P Berchialla, L Charrier, P Dalmasso, RI Comoretto

**Affiliations:** 1 Department of Public Health and Pediatrics, University of Torino, Turin, Italy; 2 Post Graduate School of Medical Statistics, University of Torino, Turin, Italy; 3 Department of Clinical and Biological Science, University of Torino, Orbassano, Italy

## Abstract

**Background:**

The impact of the COVID-19 pandemic on adolescent suicidality is still controversial. The present systematic review and meta analysis aim to summarise findings from emerging literature about prevalences of the main suicidal outcomes among children and adolescents under 19 years old, and to compare them with the pre-pandemic period.

**Methods:**

Five databases (PubMed, Embase, Scopus, CINAHL and Web of Science) were systematically searched for studies published in English from January 1st, 2020 until November 3rd, 2021, reporting prevalence for suicidal ideation (SI), suicidal behaviors (SB) and suicide (S) in the general population aged <19 during the COVID-19 pandemic. Single-study prevalence data were pooled using random-effects meta-analysis. If studies reported prevalence estimates for both pre- and during-pandemic periods, prevalence ratio (PR) comparing the two periods has been computed and pooled.

**Results:**

Sixteen observational studies were selected: 10 about SI, 9 about SB and 3 about S. During the pandemic, prevalence of SI among adolescents was 21% (95% CI 12-34%) while prevalence of SB was 3% (95% CI 1-10%). Comparing pre- and during- pandemic prevalences, a significant overall increase in SB was observed (PR 1.35; 95% CI 1.06-1.72) while the prevalence rates of SI remained substantially steady (PR 0.95; 95% CI 0.64-1.39). A narrative review on the population-based data on suicide rates suggested an escalating trend since Summer 2020, after an initial stability of the phenomenon.

**Conclusions:**

During the COVID-19 pandemic SB showed a 35% increase and suicides rates escalated after a initial stability. School closures might be involved, representing an initial protective factor for suicidality, while after the reopenings we could have assisted to a suicide rebound, especially among the most vulnerable young people.

**Key messages:**

• The topic of suicidality among young people is still affected by a cultural stigma that hampers the development of both academic research, early detection and preventive policies.

• The increase in suicidal behaviors among youths after the COVID-19 outbreak highlights a major public health issue that requires adequate supporting policies to control and prevent this phenomenon.

